# Precision Medicine in Children and Young Adults with Hematologic Malignancies and Blood Disorders: The Columbia University Experience

**DOI:** 10.3389/fped.2017.00265

**Published:** 2017-12-12

**Authors:** Lianna J. Marks, Jennifer A. Oberg, Danielle Pendrick, Anthony N. Sireci, Chana Glasser, Carrie Coval, Rebecca J. Zylber, Wendy K. Chung, Jiuhong Pang, Andrew T. Turk, Susan J. Hsiao, Mahesh M. Mansukhani, Julia L. Glade Bender, Andrew L. Kung, Maria Luisa Sulis

**Affiliations:** ^1^Department of Pediatric Oncology, Memorial Sloan Kettering Cancer Center, New York, NY, United States; ^2^Division of Pediatric Hematology, Oncology and Stem Cell Transplant, Columbia University Medical Center, New York, NY, United States; ^3^Department of Pathology and Cell Biology, Columbia University Medical Center, New York, NY, United States; ^4^Department of Pediatric Hematology/Oncology, NYU Winthrop University Medical Center, Mineola, NY, United States; ^5^Department of Pediatrics, Columbia University Medical Center, New York, NY, United States; ^6^Herbert Irving Comprehensive Cancer Center, Columbia University Medical Center, New York, NY, United States

**Keywords:** genomic, hematologic malignancies, pediatric leukemia, next-generation sequencing, targeted therapy

## Abstract

**Background:**

The advent of comprehensive genomic profiling has markedly advanced the understanding of the biology of pediatric hematological malignancies, however, its application to clinical care is still unclear. We present our experience integrating genomic data into the clinical management of children with high-risk hematologic malignancies and blood disorders and describe the broad impact that genomic profiling has in multiple aspects of patient care.

**Methods:**

The Precision in Pediatric Sequencing Program at Columbia University Medical Center instituted prospective clinical next-generation sequencing (NGS) for high-risk malignancies and blood disorders. Testing included cancer whole exome sequencing (WES) of matched tumor-normal samples or targeted sequencing of 467 cancer-associated genes, when sample adequacy was a concern, and tumor transcriptome (RNA-seq). A multidisciplinary molecular tumor board conducted interpretation of results and final tiered reports were transmitted to the electronic medical record according to patient preferences.

**Results:**

Sixty-nine samples from 56 patients with high-risk hematologic malignancies and blood disorders were sequenced. Patients carried diagnoses of myeloid malignancy (*n* = 25), lymphoid malignancy (*n* = 25), or histiocytic disorder (*n* = 6). Six patients had only constitutional WES, performed for a suspicion of an inherited predisposition for their disease. For the remaining 50 patients, tumor was sequenced with matched normal tissue when available. The mean number of somatic variants per sample was low across the different disease categories (2.85 variants/sample). Interestingly, a gene fusion was identified by RNA-seq in 58% of samples who had adequate RNA available for testing. Molecular profiling of tumor tissue led to clinically impactful findings in 90% of patients. Forty patients (80%) had at least one targetable gene variant or fusion identified in their tumor tissue; however, only seven received targeted therapy. Importantly, NGS findings contributed to the refinement of diagnosis and prognosis for 34% of patients. Known or likely pathogenic germline alterations were discovered in 24% of patients involving cancer predisposition genes in 12% of cases.

**Conclusion:**

Incorporating whole exome and transcriptome profiling of tumor and normal tissue into clinical practice is feasible, and the value that comprehensive testing provides extends beyond the ability to target-specific mutations.

## Introduction

Forty-five years ago, work published by Dr. Rowley and others provided initial evidence of the causal role of specific recurrent genetic translocations and deletions in the development of cancer, specifically hematologic malignancies (1–3). Around the same time, Drs. Sakurai and Sandburg (4) suggested that specific structural chromosomal changes observed in acute myeloid leukemia (AML) carried prognostic value and could be used in the clinical management of patients. This work revolutionized the field of oncology and created the foundation for precision medicine in oncology. Since then, newly discovered mutated genes and their pathways have been used to refine the diagnosis, prognosis, and risk stratification of patients with hematologic malignancies, ultimately leading to improvement in overall survival through carefully designed clinical trials (5, 6). With the advent of high-throughput genomic technologies that allow for comprehensive genomic profiling of most cancers, we are testing the hypothesis that knowing the genetic details of a cancer could impact clinical care and improve the health of patients. Large-scale research projects such as the Therapeutically Applicable Research to Generate Effective Treatments (TARGET) (7, 8) and the Pediatric Cancer Genome Project (PCGP) (9, 10) have provided a catalog of recurrent genetic alterations present in the most common pediatric hematologic malignancies. While the potentially transformative impact of this large genomic information can be intuited, the paucity of tools and algorithms to guide uniform clinical interpretation and therapeutic decision-making have hindered clinical adoption. Furthermore, the lack of clarity about which test or combination of tests is most cost-effective, coupled with inconsistent coverage and variable payment from insurance companies, have tempered the implementation of next-generation sequencing (NGS) molecular testing in clinical practice. Several academic medical centers have evaluated the feasibility and utility of incorporating genomic profiling in pediatric oncology practice, highlighting the potential to impact and improve clinical care as well as the challenges to broad implementation (11–13).

In 2014, the Precision in Pediatric Sequencing Program (PIPseq) at Columbia University Medical Center implemented a NGS platform into its clinical practice to profile high-risk pediatric tumors and blood disorders. Matched tumor-normal whole exome sequencing (WES), targeted sequencing of a large panel of cancer-associated genes and tumor transcriptome (RNA-seq) were utilized. A multidisciplinary pediatric molecular tumor board was established to interpret results and provide guidance to treating physicians for the application of the genomic information to clinical care. We report the results of comprehensive genomic profiling, using the PIPseq platform, for 69 samples from 56 patients with high-risk hematologic malignancies and blood disorders, and discuss the broad applicability of genomic data to multiple aspects of cancer care.

## Materials and Methods

### Patients

This is a retrospective single-center case series of 56 pediatric patients with high-risk hematologic malignancies and blood disorders treated at Columbia University Medical Center (CUMC) between January 2014 and June 2017. Twenty-eight cases have been previously reported (11). High risk was defined as having a prognosis of <50% overall survival at 5 years, having relapsed disease, being refractory to previous therapies, or having a rare hematologic disorder or unusual presentation for age. When an underlying hereditary cancer predisposition was suspected, patients and their parents were also offered constitutional WES.

Participants signed consent for constitutional or tumor-normal WES either as part of an Institutional Review Board (IRB)-approved protocol (AAAB7109) or they signed the clinical consent.^1^ Written consent for clinical WES was obtained after the risks and benefits were explained to the patient and/or caregiver, which included the potential disclosure of medically actionable secondary findings, defined as germline disease-causing mutations unrelated to the condition for which sequencing was being performed. Patients could opt-in/opt-out of learning secondary findings and/or having these results appearing in the electronic medical record (EMR), having their samples and/or data stored for future research, either with or without identifiers, and future contact. Results not reported included carrier status, variants of uncertain significance (VOUS) in secondary findings except as related to cancer, and mutations related to adult-onset conditions for which the genetic link is unclear or for which no known intervention is of proven benefit. Samples were obtained in the course of clinical care and either prospectively banked for future molecular testing (IRB-AAAB7109) or freshly collected at the time of relapse. IRB approval was obtained for this retrospective analysis of de-identified patient and clinical genomics data (IRB AAAQ8170 and AAAP1200).

### Clinical Sequencing and Analysis

Nucleic acid preparation and high-throughput sequencing were performed following previously described standard protocols in the Laboratory of Personalized Genomic Medicine at CUMC (11). Sequencing was performed on fresh or frozen bone marrow or peripheral blood containing at least 50% lesional cells. Flow-sorting was used to either purify or enrich tumoral cells in the event of mixed chimerism, in patients who had received an allogeneic stem cell transplant, or when the amount of tumoral cells was below the threshold. Fresh or formalin fixed paraffin embedded tissue was used in cases of organ infiltration or chloroma. Three CLIA-certified, College of American Pathologists and New York State Department of Health approved assays were used. WES of tumor and normal tissue (buccal swab or peripheral blood) and RNA sequencing of tumor tissue was performed when possible. DNA sequencing of 467 cancer-associated genes (Columbia Comprehensive Cancer Panel, CCCP) was used when available tumoral or normal tissue was inadequate. Constitutional WES was performed when a constitutional disease was suspected on samples from both patients and parents (trio).

Whole exome and transcriptome libraries were prepared as previously described (11) and sequenced on a HiSeq2500 using paired-end 125-cycle x2sequencing. Variant calls were independently made on tumor and germline material. Variants were subject to filtering. In normal DNA, variants were passed through a “reference range filter” of cancer predisposition genes, genes relevant to pharmacogenomics, and variants relevant to patient care; a “reportable range filter” which includes COSMIC (cosmic70 provided by Annovar) variants in the patient’s mutation report file and variants in genes recommended by American College of Medical Genetics (ACMG) for the reporting of secondary findings (14); as well as a frequency filter, which includes variants whose minor allele frequency in the 1,000 genomes (phase 1, version 3, release date 11/23/2010) is less than 1%. Somatic mutations in the tumor were identified by subtracting all variants called in normal tissue (output at minor allelic fraction of ≥5%) from variants called in the tumor (output at a minor allelic fraction of ≥10%). The approach maximized the number of variants output to minimize the likelihood of filtering out actionable mutations prior to molecular tumor board discussion. Copy number variation (CNV) was determined from the WES data and fusion transcripts and relative gene expression were identified from the RNA-seq data. Tumor and normal DNA and tumor RNA sequence analysis was previously described (11). Quality statistics for tumor-normal WES are presented in Table S1 in Supplementary Material.

### Data Interpretation and Reporting

**Figure 1:**
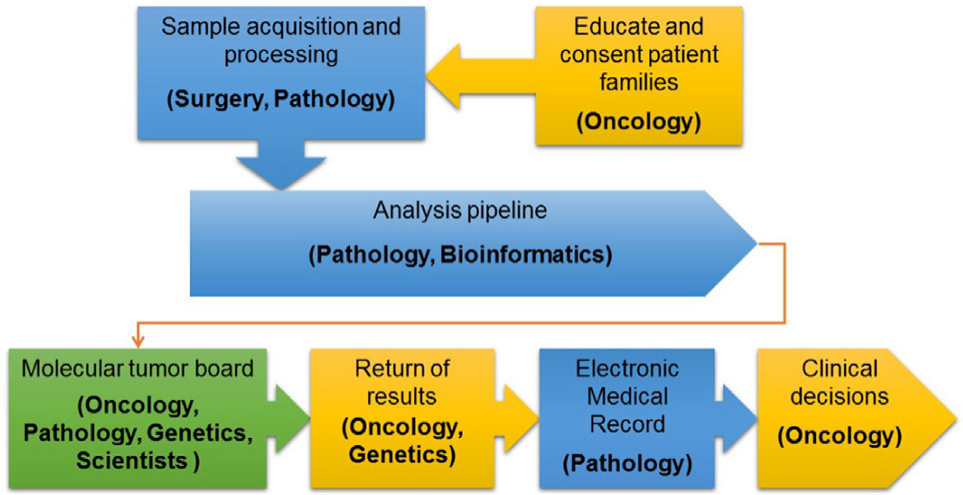
Overview of the precision in pediatric sequencing program clinical workflow.

After initial review by a molecular pathologist, interpretation of each tumor-normal WES and RNA-seq results was conducted in a weekly multidisciplinary molecular tumor board. The tumor board included members from pediatric oncology, molecular pathology, cytogenetics, medical genetics, cancer biology, and bioinformatics. Clinical relevance of variant calls, CNV, and fusions was based on published laboratory data documenting gain or loss of function mutations in known oncogenes and tumor suppressor genes, respectively or predicted by *in silico* algorithms or determined based on previously reported gene fusions or gene fusions expected to have the same effect as previously reported fusions involving one of the partner genes. Additionally, factors such as recurrence of the identified variant and allelic fraction were considered when addressing clinical significance (11). The final clinical report provided a tiered description of somatic mutations based on disease-association and evidence for clinical actionability. Tier 1 included known, tumor type-specific actionable somatic mutations; Tier 2 included somatic mutations in targetable pathways, actionable somatic mutations in other tumor types and somatic mutations in well-established cancer genes; Tier 3 included other somatic mutations in cancer genes; and Tier 4 included somatic VOUS. Reporting of germline variants was based on whether they were known to be pathogenic, recommended by the ACMG for reporting of secondary findings (14) or VOUS in cancer genes or genes potentially important for patient care. Accession number for all genes and fusions reported are provided in Table S2 in Supplementary Material. Reports were sent to the primary oncologists and posted to the EMR in accordance with patient opt-in/opt-out preferences selected at the time of informed consent. An overview of the PIPseq workflow is presented in Figure 1. Datasets are available through cBioPortal for Cancer Genomics^2^ (15, 16).

For the purposes of this study, we defined *clinically impactful findings* as any molecular test result that, when integrated with a patient’s history, symptoms, and other clinical findings, could lead to a change in the assessment or management of the patient. This definition is in agreement with the recommendations provided by the Association for Molecular Pathology to evaluate the clinical utility of a molecular test (17). To evaluate the potential clinical utility of tumor and germline alterations, these clinically meaningful results were subcategorized into the following five categories: (1) diagnostic; (2) prognostic; (3) identification of a therapeutic target; (4) other clinically impactful information, including pharmacogenomics or findings that resulted in a significant refinement of a therapeutic plan; and (5) recommendations for health maintenance interventions or genetic counseling for the patient and other at-risk family members. Genetic alterations were considered “targetable” if: (1) an FDA approved drug or experimental drug was available that inhibited the target directly or inhibited its downstream signaling pathway; (2) there was pre-clinical evidence to support efficient targeting of the aberrant function of the mutated gene and/or potential clinical benefit; and (3) there was information on age-appropriate dosing. “Targetable” mutations were further categorized using a five-tier system, previously described (18, 19). This tiering system evaluates the potential clinical benefit of targeting an altered gene based on published preclinical and clinical data.

## Results

**Figure 2:**
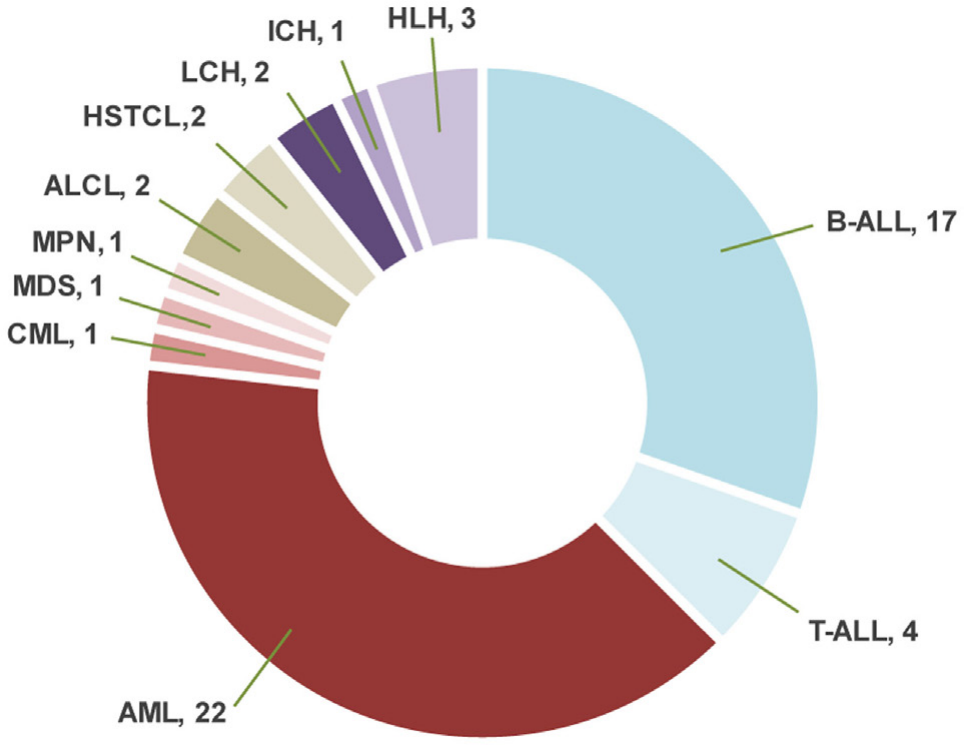
Distribution of the diagnoses in patients with hematologic malignancies and blood disorders. ALL, acute lymphoblastic leukemia; AML, acute myeloid leukemia; CML, chronic myeloid leukemia; MDS, myelodysplastic syndrome; MPN, myeloproliferative neoplasm; ALCL, anaplastic large cell lymphoma; HSTCL, hepatosplenic T-cell lymphoma; LCH, Langerhans cell histiocytosis; ICH, indeterminate cell histiocytosis; HLH, hemophagocytic lymphohistiocytosis.

Sixty-nine samples obtained from 56 patients underwent molecular profiling (Table 1). Twenty-one patients (37.5%) had acute lymphoblastic leukemia (ALL), 25 patients (44.7%) had a myeloid neoplasm, 4 patients (7.1%) had a lymphoma, and 6 patients (10.7%) had a histiocytic disorder (Figure 2). The most common reasons for requesting molecular profiling were relapsed or refractory disease, suboptimal response to initial chemotherapy, high-risk features at presentation, or a rare tumor with no standard of care therapy. WES and RNA-seq were performed on 43/69 samples (62.3%); WES was only performed on 12/69 samples (17.4%) and targeted panel sequencing was performed on 6 samples (8.7%). Six patients had only constitutional WES performed for suspicion of an underlying predisposition to cancer or immunodeficiency; two patients had both tumor and constitutional WES performed following findings on tumor profiling concerning for either a germline variant or a cancer predisposing condition. For nine patients, serial samples, either paired diagnostic and relapse samples, or sequential relapse samples were available for genomic profiling and analysis of clonal evolution. Over 150- and 500-fold average coverage was achieved by WES and targeted capture sequencing, respectively, with >98% of the coding sequencing having at least tenfold coverage. Of the 56 cases, 39 were consented using the clinical tumor-normal WES consent form. Only six (15%) opted out of learning secondary findings; 17% opted out of having secondary findings in their medical records and all patients agreed to have leftover samples stored for future research. The median turnaround time from sample submission to presentation at the molecular tumor board was 40 days. Patients and their families were not charged for sequencing or analysis. Testing that was not reimbursed by insurance was paid for using institutional and philanthropic funds.

**Table 1:** Patient demographics and sample characteristics.

	No. (%) by diagnostic category			
	Lymphoid (*n* = 21)	Myeloid (*n* = 25)	Lymphoma (*n* = 4)	Histiocytic disorder (*n* = 6)
**Gender**				
Male	15 (71)	12 (48)	2 (50)	3 (50)
Female	6 (29)	13 (52)	2 (50)	3 (50)

**Age, years**				
Mean (median)	9.5 (8.0)	9.9 (10.0)	13.8 (16)	5.7 (2)
Samples tested	28	30	5	6
Primary disease	13 (46.5)	9 (30)	3 (60)	3 (50)
Relapse/refractory	14 (50)	18 (60)	1 (20)	0 (0)
Germline^a^	1 (3.5)	3 (10)	1 (20)	3 (50)

**Platform**				
Tumor-normal WES with transcriptome	23 (82)	17 (57)	2 (40)	1 (17)
Tumor-normal WES	3 (10)	7 (23)	2 (40)	0 (0)
Constitutional WES	1 (4)	3 (10)	1 (20)	3 (50)
Targeted cancer panel	1 (4)	3 (10)	0 (0)	2 (33)

*WES, whole exome sequencing*.

*^a^Indicates sample used for constitutional WES*.

### Genomic Alterations and Clinical Impact in Patients with ALL

**Figure 3:**
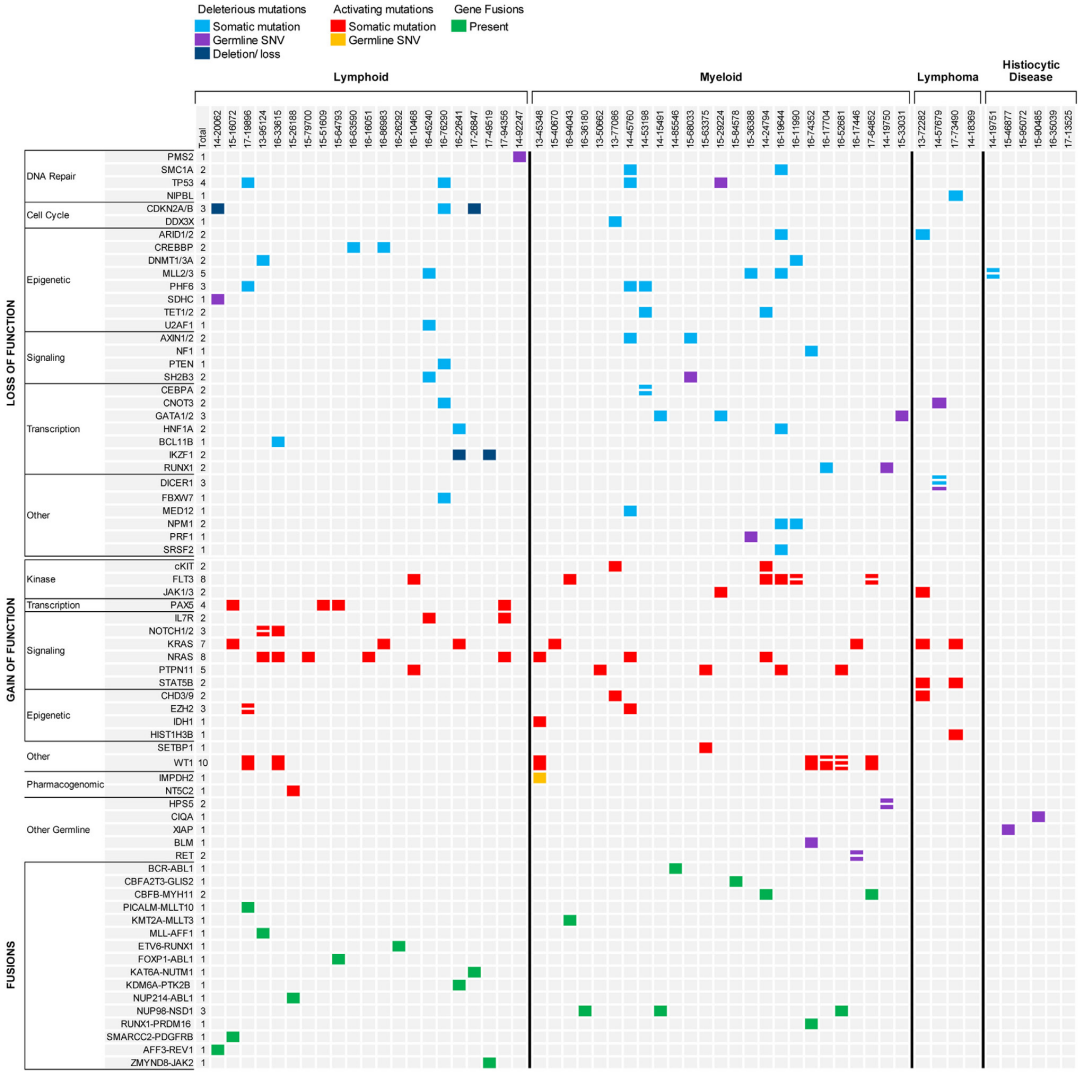
Summary of results for hematologic malignancies and blood disorders sequenced through the precision in pediatric sequencing program. Only findings with biological and clinical significance are represented.

Molecular profiling was performed on 27 bone marrow or peripheral blood samples obtained from 20 patients with ALL (mean age, 9.5 years; median age, 8.0 years; range, 1.5–23.9 years) (Table 1). Three patients had T-ALL and 17 had B-ALL (Figure 2). Sequencing was performed at time of diagnosis in 10 patients and at time of relapse in the remaining 10 patients. Paired diagnostic and relapsed samples were available for sequencing from three patients while serial relapse and refractory samples were sequenced from two other patients. One patient with T-ALL had constitutional WES only, because of strong family history of cancer. The mean mutational load across patients was 705.2 variants (SD = 1,735.6; median = 93.0; range = 30–5,950). After filtering, 81 somatic variants were identified in 34 genes, most of them classified as Tier 2 (53*/*81, 65.4%) (Figure 3; Table S2 in Supplementary Material). The mean number of somatic variants per sample, after filtering, was 3.0 (median = 2.0; range = 0–13) and was not significantly different between relapsed and diagnostic samples (3.71 vs. 2.23, *p* = 0.19). Signaling pathways most frequently, aberrantly activated were the RAS pathway (*NRAS, n* = 8; *KRAS, n* = 4; *PTPN11, n* = 1) and the JAK-STAT pathway (*IL7R, n* = 2; *SH2B3, n* = 1; *JAK3, n* = 1). As expected, several transcription factors critical for the normal development and differentiation of B- and T-cells were also recurrently mutated, such as *PAX5* (*n* = 4) and *NOTCH1* (*n* = 5). Additionally, three B-ALL samples carried mutations in genes responsible for resistance to chemotherapy (*CREBBP, n* = 2; *NT5C2, n* = 1). Analysis of CNVs revealed loss of *CDKN2A* in two samples and *IKZF1* in three samples.

Nine unique fusions were identified using RNA-seq in nine patients. Fusions were conserved in paired diagnostic and relapse samples, as well as, in serial relapsed and refractory samples and were the only driver alteration identified in 4/9 cases. Gene fusions involved a kinase in four cases and a transcription factor in the remaining five cases, likely causing constitutive activation of a signaling pathway and aberrant cell differentiation, respectively (Figure 3). Analysis of RNA-seq data also allowed the recognition of a *BCR-ABL1*-like ALL expression pattern in a patient with relapse ALL and no identifiable driver mutation. Further testing led to the identification of a *NUP214-ABL1* fusion by real-time polymerase chain reaction (RT-PCR) (20) and subsequent addition of dasatinib to conventional chemotherapy. Interestingly, the analytic pipeline failed to identify the *NUP214-ABL1* fusion despite adequate coverage and good quality of the specimen. Further analysis using three different pipelines, as well as, repeat RNA sequencing on a subsequent refractory sample, also failed to recover the fusion. We speculate that the fusion might have been present in a smaller subclone below the level of detection of current sequencing technologies.

**Figure 4:**
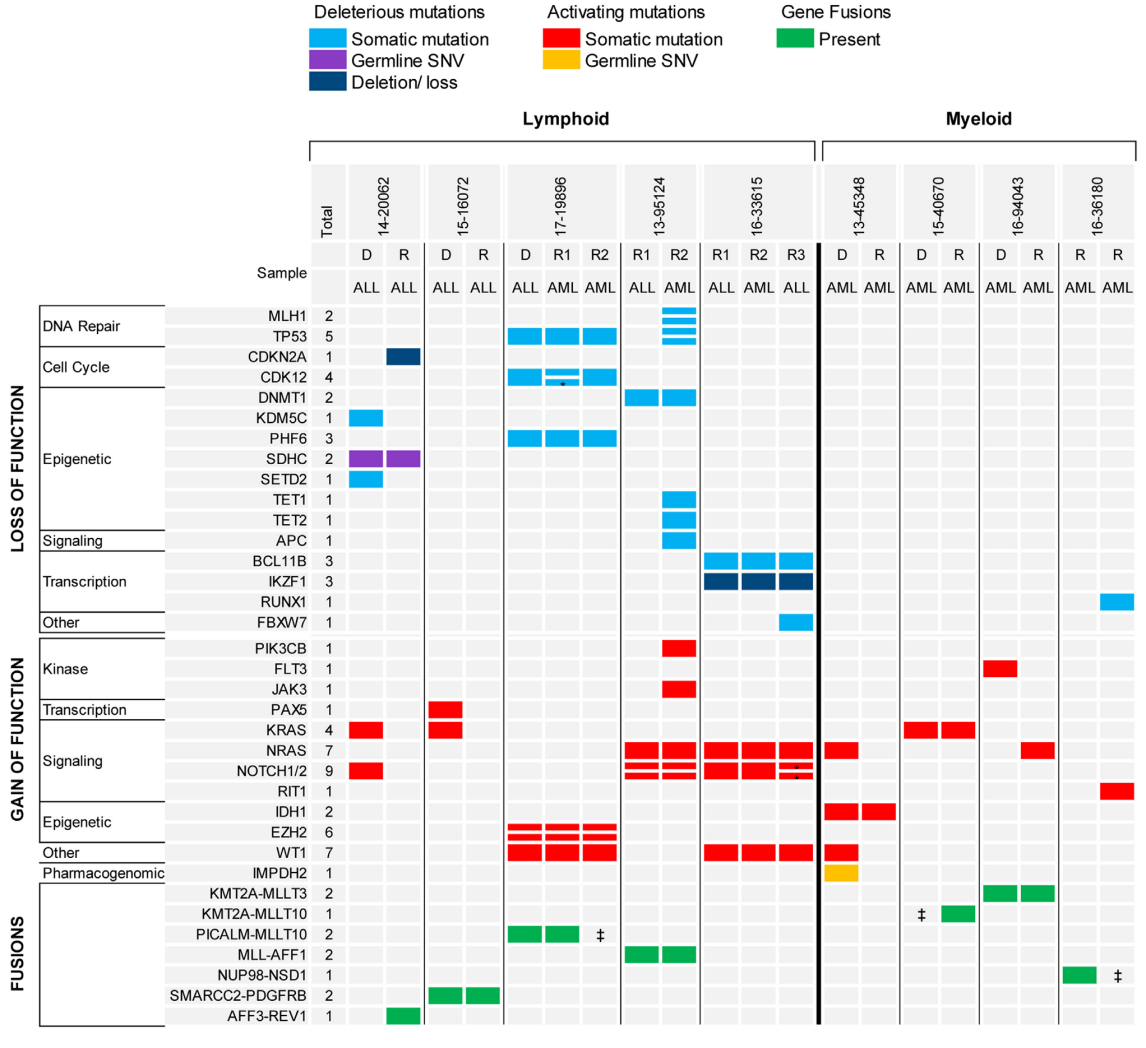
Recurrent genomic alterations in paired diagnostic-relapse and serial relapsed samples. *Indicates same gene, different variant. ^‡^Indicates RNA-seq not done, fusion detected by FISH.

The analysis of serial samples from five patients was particularly interesting (Figure 4). Three patients had B-ALL and two patients had early thymocyte precursor (ETP) ALL. In two patients with B-ALL, the gene fusion present at diagnosis was the only genetic alteration maintained at relapse, suggesting that the fusion represented the main driver present in the founding clone. Conversely, none of the somatic mutations found in the diagnostic samples were retained in the relapsed samples, possibly indicating the eradication of several subclones by the initial therapy. The third patient with B-ALL had relapsed *MLL*-rearranged infant ALL that switched to *MLL*-rearranged AML at the completion of one cycle of blinatumomab. In this case, the *KMT2A-AFF1* fusion and all somatic gene mutations present in the relapse ALL sample were shared in the AML sample, but the AML sample had also acquired several new genetic alterations, some in genes more commonly mutated in myeloid diseases (e.g., *TET1* and *TET2*). The two patients with ETP also experienced a relapse with phenotypic switch to a myeloid, monocytic leukemia. The first patient presented with relapsed ETP and, while receiving the first cycle of re-induction chemotherapy with standard chemotherapy (vincristine, prednisone, PEG-aspargase, and doxorubicin) plus everolimus, the leukemia evolved into myelomonocytic leukemia with loss of T-cell markers and expression of myeloid markers. The two samples (ETP-ALL and AML) carried identical genetic alterations. The patient experienced a second ETP-ALL relapse after stem cell transplant. In this sample, the activating mutation found in *NOTCH1* at diagnosis was lost, but two new *NOTCH1* mutations were acquired together with a loss of function mutation in *FBXW7*, suggesting that the leukemia was “addicted” to activated NOTCH1 signaling. Interestingly, while receiving palliative chemotherapy with a gamma-secretase inhibitor, the ETP-ALL again switched immunophenotype and evolved into AML. The second patient with ETP relapsed during standard chemotherapy as an isolated thymic granulocytic sarcoma. The diagnostic and the two relapse myeloid samples in this case also shared identical genetic alterations with both the same gene fusion, as well as, the somatic gene mutations identified in all samples.

Approximately 37% of gene variants and copy number losses identified by tumor-normal WES were considered targetable (Table 2). However, only 2 of the 15 patients (13%) carrying targetable mutations in their tumor received targeted therapy. In contrast, 60% of the transcriptome findings were targetable and 4/6 patients whose leukemia carried targetable gene fusions received effective targeted therapy (Table 2). For example, one patient with a second relapse of ALL occurring post-transplant achieved complete remission with no minimal residual disease 2 months after receiving standard maintenance therapy with dasatinib for a *SMARCC2-PDGFRB* fusion. Overall, 19/20 patients with ALL carried at least one targetable alteration.

**Table 2:** Tiered classification of potentially targetable somatic mutation for treatment planning.

PIP ID	Diagnosis	Target alteration	Mutation (change)	Potential target therapy
**Tier 1 (data demonstrating clinical benefit—same tumor type, same gene)**					

13-95124^a^^,^^c^	B-ALL	*KMT2A-AFF1*	Fusion	DOT1L inhibitor (21, 22)
16-33615^a^	T-ALL	*NOTCH1*	c.4775C > T (p.F1592S); c.4898G > A (p.R1633H); c.4799T > C (p.L1600P)	Gamma-secretase inhibitor^b^ (23)
14-24794^a^^,^^c^	AML	*c-KIT; FLT3; NRAS*	c.2446G > C (p.D816H); c.2505T > G (p.D835E); c.183A > C (p.Q61H)	TKI (24); FLT3 inhibitor (25); MEK inhibitor (26)
13-77086^c^	AML	*c-KIT*	c.1965T > G (p.Asn655Lys)	TKI^b^ (24)
16-11990^a^	AML	*FLT3*	c.2028C > G (p.N676K) and c.2503G > T (p.D835Y)	FLT3 inhibitor (25)
16-19644^a^	AML	*FLT3*	c.1987A > C (p.K663Q)	FLT3 inhibitor (25)
17-64852^a^	AML	*FLT3*	c.2542G > C (p.A848P) and c.1774G > T (p.V592F)	FLT3 inhibitor (25)
13-45348^c^	AML	*IDH1; NRAS*	c.394C > T (p.R132C); c.38G > C (p.G13A)	IDH inhibitor (27); MEK inhibitor (26)
16-94043	AML	*NRAS; FLT3; KMT2A-MLLT3*	c.183A > C (p.Gln61His); c.2508_2510delCAT (p.I836del); fusion	MEK inhibitor (26); FLT3 inhibitor (25); DOT1L inhibitor (21, 22)
15-40670	AML	*KRAS; KMT2A-MLLT10*	c.38G > A (p.G13D); fusion	MEK inhibitor (26), DOT1L inhibitor (21, 22)
16-17446^a^	AML	*KRAS*	c.183A > C (p.Q61H)	MEK inhibitor (26)
14-45760^c^	AML	*NRAS*	c.38G > A (p.G13D)	MEK inhibitor (26)

**Tier 2 (data demonstrating clinical benefit—different tumor type, same gene)**					

13-95124^a^^,^^c^	B-ALL/AML	*TET2*	c.1805G > A (p.R602H)	Hypomethylating agent (28)
17-26847	B-ALL	*CDKN2A* deletion	Copy number change	CDK4/6 inhibitor (29)
16-10468^a^	B-ALL	*FLT3*	c.2028C > A (p.N676K)	FLT3 inhibitor (25)
14-20062^a^^,^^c^	B-ALL	*KRAS; NOTCH1*	c.183A > T (p.Q61H); c.3047G > T (p.S1016I)	MEK inhibitor (26); Gamma-secretase inhibitor (23)
15-16072^a^^,^^c^	B-ALL	*KRAS*	c.38G > A (p.G13D)	MEK inhibitor (26)
16-22641^a^	B-ALL	*KRAS*	c.35G > A (p.G12D)	MEK inhibitor (26)
16-86983	B-ALL	*KRAS*	c.38G > A (p.G13D)	MEK inhibitor (26)
13-95124^a^^,^^c^	B-ALL	*NRAS; PIK3CB*	c.182A > G (p.Q61R); c.754G > A (p.G252R)	MEK inhibitor (26); PI3K inhibitor (30)
15-79700^c^	B-ALL	*NRAS*	c.183A > T (p.Q61H)	MEK inhibitor (26)
16-16051	B-ALL	*NRAS*	c.38G > A (p.G13D)	MEK inhibitor (26)
17-94356^a^	B-ALL	*NRAS; CDKN2A* deletion	c.35G > A (p.G12D); copy number change	MEK inhibitor (26); CDK4/6 inhibitor (29)
15-51609	B-ALL	*TSC2*	c.344G > A (p.R115H)	mTOR inhibitor (31)
16-33615^a^	T-ALL	*NRAS*	c.34G > A (p.G12S)	MEK inhibitor (26)
16-76290^a^	T-ALL	*PTEN; CDKN2A* deletion	c.702_703insC (p.E235fs); copy number change	mTOR inhibitor (32, 33); CDK4/6 inhibitor (29)
16-74352	AML	*NF1*	c.2033dup (p.I679Dfs)	mTOR inhibitor (34)
14-53198^c^	AML	*TET2*	c.1156G > T (p.V386L)	Hypomethylating agent (28)
14-24794^a^^,^^c^	AML	*TET2*	c.3663delT (p.C1221Wfs);	Hypomethylating agent (28)
17-73490^a^	T-cell lymphoma	*KRAS*	c.35G > T (p.G12V)	MEK inhibitor (26)
13-72282^a^^,^^c^	T-cell lymphoma	*KRAS*	c.40G > A (p.V14I); c.3076A > G (p.K1026E)	MEK inhibitor (26)

**Tier 3 (published, presented, in press pre-clinical data demonstrating benefit—same tumor type, same gene)**				

15-64793^c^	B-ALL	*FOXP1-ABL1*	Fusion	TKI^b^ (20, 35)
16-45240	B-ALL	*IL7R; SH2B3*	c.727_728insGGTGCT (p.L242_L243insRC); c.1248dupG (p.S417Vfs)	JAK inhibitor^b^ (20, 36)
17-94356^a^	B-ALL	*IL7R*	c.731_732insGGGGTCGGGGTGCTT (p.T244_I245insGSGCF)	JAK inhibitor (20, 36)
15-26188^c^	B-ALL	*NUP214-ABL1*	Fusion	TKI^b^ (20)
16-10468^a^	B-ALL	*PTPN11*	c.417G > C (p.E139D)	MEK inhibitor (37)
15-16072^a^^,^^c^	B-ALL	*SMARCC2-PDGFRB*	Fusion	TKI^b^ (20)
17-49519^a^	B-ALL	*ZMYND8-JAK2*	Fusion	JAK inhibitor^b^ (20, 36)
15-29224^c^	AML	*JAK3*	c.1718C > T (p.A573V)	JAK inhibitor (38)
14-15491^c^	AML	*NUP98-NSD1*	Fusion	DOT1L inhibitor (39)
16-36180^a^	AML	*NUP98-NSD1*	Fusion	DOT1L inhibitor (39)
16-52681^a^	AML	*NUP98-NSD1; PTPN11*	Fusion; c.226G > A (p.E76K)	DOT1L inhibitor (39); MEK inhibitor (37, 40, 41)
13-50662^c^	AML	*PTPN11*	c.1508G > T (p.G503V)	MEK inhibitor (37, 40, 41)
15-63375^c^	AML	*PTPN11*	c.181G > T (p.D61Y)	MEK inhibitor (37, 40, 41)
16-19644^a^	AML	*PTPN11*	c.133G > C (p.V45L)	MEK inhibitor (37, 40, 41)
17-73490^a^	HSTCL	*STAT5B*	c.2135T > A (p.V712E)	JAK inhibitor (42); STAT inhibitor (43)
13-72282^a^^,^^c^	HSTCL	*STAT5B*	c.2110A > C (p.1704L)	JAK inhibitor (42); STAT inhibitor (43)

**Tier 4 (published, presented, in press pre-clinical data demonstrating benefit—different tumor type, same gene)**				

13-95124^a^	B-ALL/AML	*JAK3*	c.2869G > A (p.V957M)	JAK inhibitor (38)
14-20062^a^^,^^c^	B-ALL	*SETD2*	c.4405delA (p.L1467fs)	WEE1 inhibitor (44)
16-26292	B-ALL	*SETD2*	Copy number change	WEE1 inhibitor (44)
17-19896	T-ALL	*PICALM-MLLT10*	Fusion	DOT1L inhibitor (45)
15-36388^c^	AML	*MLL3 (KMT2C)*	c.2110G > T (p.E704X)	BET inhibitor (46)

**Tier 5 (anything else the molecular tumor board thought was sufficient to qualify for treatment planning)**				

16-22641^a^	B-ALL	*KDM6A-PTK2B*	Fusion	FAK inhibitor (47)

*B-ALL, B-cell acute lymphoblastic leukemia; T-ALL, T-cell acute lymphoblastic leukemia; AML, acute myeloid leukemia; MPN, myeloproliferative neoplasm*.

*^a^Case listed more than once*.

*^b^Targeted therapy received*.

*^c^Case previously reported (11)*.

The clinical impact of the genomic alterations identified in ALL through NGS goes well beyond the findings of targetable lesions, frequently in ways that are not easily quantifiable. In four patients, identification of an *IKZF1* deletion, *FOX1-ABL1* fusion, and a specific RNA-seq profile led to a refined diagnosis of *BCR-ABL* like ALL and related poor prognosis (20). In one patient with relapsed B-ALL, the finding of a *NT5C2* mutation, responsible for resistance to nucleoside analogs (48, 49), had clear implications for subsequent therapy (Table 3). Last, for the two patients with ETP that evolved to AML, the identification of shared DNA and RNA alterations between the ALL and AML samples led to the conclusion that the AML was not a second cancer or a secondary AML, but rather a immunophenotypic switch of the same leukemic clone; this assessment clearly impacted the subsequent clinical management of both patients.

**Table 3:** Clinical utility beyond targetable somatic mutations.

PIP ID	Diagnosis	Alteration	Mutation (change)	Clinical utility	Implication
**Sequence mutations**					

15-26188^a^^,^^b^	B-ALL	*NT5C2*	c.1219G > T (p.D407Y)	Pharmacogenomic	Affects therapy
16-11990^a^	AML	*NPM1*	c.860_863dupTCTG (p.W288fs*12)	Prognostic	Good prognosis
16-19644^a^	AML	*NPM1*	c.860_864dup (p.W288fs)	Prognostic	Good prognosis
13-45348^a^^,^^b^	AML	*IDH1*	c.394C > T (p.R132C)	Diagnostic	Contributed to establishing the diagnosis of Maffucci syndrome
14-53198^a^^,^^b^	AML	*CEBPA*	c.939_940insAAG (p.K313_V314insK); c.326_327insC (p.P109fs)	Prognostic	Good prognosis
15-63375^a^^,^^b^	AML	*PTPN11; SETBP1*	c.181G > T (p.D61Y); c.2602G > A (p.D868N)	Diagnostic	Contributed to establishing the diagnosis of AML derived from JMML
13-72282^a^^,^^b^	HSTCL	*STAT5B*	c.2110A > C (p.I704L)	Diagnostic	Gamma-delta T-cell lymphoma
17-73490^a^	HSTCL	*STAT5B*	c.2135T > A (p.V712E)	Diagnostic	Gamma-delta T-cell lymphoma

**Transcriptome analysis**					

16-26292^a^	B-ALL	*ETV6-RUNX1*	Fusion	Prognostic	Good prognosis
15-84578^b^	AMKL	*CBFA2T3-GLIS2*	Fusion	Diagnostic; prognostic	Contributed to establishing the diagnosis of AMKL; poor prognosis
14-24794^a^^,^^b^	AML	*CBFB-MYH11*	Fusion	Prognostic	Good prognosis
17-64852^a^	AML	*CBFB-MYH11*	Fusion	Prognostic	Good prognosis
14-15491^a^^,^^b^	AML	*NUP98-NSD1*	Fusion	Prognostic	Poor prognosis
16-36180^a^	AML	*NUP98-NSD1*	Fusion	Prognostic	Poor prognosis
16-52681^a^	AML	*NUP98-NSD1*	Fusion	Prognostic	Poor prognosis

**Copy number variation**					

16-22641^a^	B-ALL	*IKZF1* deletion	Copy number change	Prognostic	Poor prognosis
17-49519^a^	B-ALL	*IKZF1* deletion	Copy number change	Prognostic	Poor prognosis

*B-ALL, B-cell acute lymphoblastic leukemia; AML, acute myeloid leukemia; AMKL, acute megakaryoblastic leukemia; JMML, juvenile myelomonocytic leukemia; HSTCL, hepatosplenic T-cell lymphoma*.

*^a^Case listed more than once*.

*^b^Case previously reported (11)*.

### Genomic Alterations and Clinical Impact in Patients with Myeloid Neoplasms

Twenty-eight bone marrow, peripheral blood, or chloroma samples, obtained from 23 patients with myeloid neoplasms, underwent molecular profiling (mean age, 9.9 years; median age, 10.0 years; range, 0.9–23.9 years) (Table 1). Sequencing was performed at diagnosis in 8 patients and at time of relapse in 15 patients. Paired diagnostic and relapsed samples were available for sequencing in two patients, while serial relapse and refractory samples were sequenced from two other patients. About 1 patient had relapsed chronic myeloid leukemia (CML), 1 patient had a myeloproliferative neoplasm (MPN), and the remaining 21 patients had AML (Figure 2). Among the 21 patients with AML, 10 were classified as AML with recurrent genetic abnormalities (50) [biallelic mutation of *CEBPA, n* = 1; *NPM1, n* = 2; *KMT2A* rearrangement, *n* = 2; inv16 (p13.1q22), *n* = 2; t(8;21)(q22;q22.1), *n* = 1; *RUNX1* mutation, *n* = 2], one patient had AML associated with Down syndrome, one patient had AML with myelodysplasia related changes, and the remaining nine patients had AML not otherwise specified. In two patients suspected to have an underlying genetic predisposition to cancer, only constitutional WES was performed. Constitutional WES was also performed in one patient after WES findings on the tumor-normal samples raised the possibility of germline genetic alterations.

The mean mutational load across patients was 101.7 variants (SD = 89.74; median = 79.0; range = 14–457). After filtering, 77 somatic variants were identified in 48 genes, most of them classified as Tier 2 (69%) (Figure 3; Table S2 in Supplementary Material). All patients had at least one genomic alteration. The mean number of somatic variants per sample after filtering was 2.85 (median = 2.0; range = 0–13) with similar mean number in diagnostic and relapsed samples (3.6 vs. 2.4, *p* = 0.30). *WT1* and *FLT3* were the most commonly mutated genes (*n* = 8 and *n* = 7, respectively) as well as members of the RAS pathway (*PTPN11, n* = 5; *NRAS, n* = 4; and *KRAS, n* = 3). As expected, gene mutations commonly seen in adult AML, such as in *TET2, DNMT3A, ASXL1, EZH2*, and in members of the spliceosome machinery (50, 51), were only rarely encountered in our cohort. Internal tandem duplications of the *FLT3* gene are not easily identifiable through NGS, however, the presence of FLT3ITD was confirmed in two AML samples through PCR. RNA sequencing identified seven unique gene fusions in 12 samples from 10 patients, of which four fusions were the product of cryptic chromosomal inversions and translocations not identified by traditional chromosomal banding. Unlike what was observed in the ALL samples, gene fusions identified in the AML samples never involved kinases, but rather involved transcription factors or co-activators and co-repressors of transcriptional complexes likely critical for the proper development and differentiation of hematopoietic precursors. These fusions are considered class II mutations in the classical “two-hit” model proposed more than a decade ago by Gilliland (52).

The analysis of the two diagnostic-relapse pairs and the serial relapsed samples showed a simple model of clonal evolution, different from the pattern of clonal evolution described in adult AML (Figure 4). One diagnostic AML sample, characterized by a *KMT2A* rearrangement, lost the *FLT3* TKD mutation and gained a *NRAS* mutation at relapse; one AML relapsed sample with a *NUP98-NSD1* fusion, gained only a new mutation in *Runx1* and *RIT1* in a subsequent refractory sample. In another patient with AML, which likely evolved from a myelodysplastic syndrome (MDS), the MDS and secondary AML samples shared a mutation in *IDH1* and the AML gained additional mutations only in *NRAS* and *WT1* genes. These patterns of clonal evolution are consistent with what was previously described in the TARGET cohort of paired diagnostic and relapsed samples in pediatric AML (8).

Twenty-seven of the 77 variants identified were considered targetable (35%) (Table 2) with 19/23 patients (82%) carrying at least one targetable genetic variant. However, only one patient received targeted therapy. The patient, with a previously known t(8;21)(q22;q22.1) AML and found at relapse to carry an activating mutation of c-*KIT*, received targeted therapy with imatinib; the total peripheral white blood cell count normalized and the blast count significantly decreased, allowing a sustained control of disease for ~9 months until imatinib was discontinued due to toxicity. Three of the seven fusions were also considered targetable, however, none of the patients received targeted therapy. Importantly, 2/77 somatic variants and 5/7 gene fusions carried prognostic significance, in one case (*CBFA2T3-GLIS2* fusion) leading to the decision to proceed to stem cell transplant as consolidation therapy. Finally, findings from WES/RNA-seq led to the correct diagnosis in two cases. In one child, the finding of mutated *PTPN11* and *SETBP1*, together with cytogenetics and clinical findings, led to the correct diagnosis of AML evolved from JMML rather than *de novo* AML. In a second patient, the initial diagnosis of AML with minimal differentiation was corrected to megakaryoblastic leukemia following the finding on *CBFA2T3-GLIS2* fusion by RNA-seq (Table 3). Overall, the findings obtained by NGS of myeloid leukemia samples provided clinically relevant information in 20 patients (86%).

### Genomic Alterations and Clinical Impact in Patients with Lymphoma

Two samples from two patients with hepatosplenic gamma-delta T-cell lymphoma (HSTCL) and two samples from two patients with anaplastic large cell lymphoma (ALCL) were sequenced (mean age, 13.8 years; median age, 16.0 years; range, 5–18 years) (Table 1; Figure 2). One patient also had constitutional WES because of family history of cancer. Tumor tissue was obtained at diagnosis in two cases and at relapse in the remaining two. The mean mutational load was 248.5 variants (SD = 374.1; median = 75.0; range = 36–808). After filtering, 12 somatic variants were identified in 9 genes (Figure 3; Table S2 in Supplementary Material). The mean number of somatic variants per sample was 3.0 (median = 2.5; range = 0–5). The two HSTCL samples shared activating mutations in *KRAS* and *STAT5B*, as well as, in genes involved in the epigenetic modulation of transcription. Importantly, mutations in *KRAS* and *STAT5B* were considered targetable, although neither patient received a targeted therapy (Table 2). The finding of a *STAT5B* mutation in one of the two patients, together with the cytogenetic finding of isochromosome 7q, was particularly important in confirming the diagnosis of HSTCL as the patient was previously erroneously diagnosed and treated for T-ALL (Table 3).

### Genomic Alterations and Clinical Impact in Patients with Histiocytic Disorders

Two patients with Langerhans cell histiocytosis and one patient with extensive cutaneous and mucosal involvement by indeterminate cell histiocytosis had tumor samples sequenced (Table 1; Figure 2). Tumor-normal WES was done in one sample and targeted panel sequencing was performed in the remaining two samples. Neither variants commonly identified in histiocytic disorders (53), nor variants of significance in cancer related genes were identified in these samples. Three patients presented with hemophagocytic lymphohistiocytosis (HLH) and underwent constitutional WES to investigate an underlying inherited immunodeficiency disorder or familial HLH (54) (Table 1; Figure 2). One patient with several congenital abnormalities was referred to our institution for management of HLH and stem cell transplantation. However, he was later found to have a compound germline mutation in *MLL2* leading to the diagnosis of Kabuki syndrome (55) and stem cell transplant was averted. The remaining two children with HLH were found to carry a germline mutation in *C1QA* and *XIAP*, respectively and stem cell transplantation was recommended for both children (Table 4).

**Table 4:** Clinically impactful germline mutations.

PIP ID	Diagnosis	*Gene* mutation	Mutation classification	Variant prediction	Inherited/*de novo*	Clinical utility	Clinical diagnosis/implication	Known
14-19750^c^	AML	*RUNX1* c.806-2A > G, r.Spl?	Heterozygous, splice site	Pathogenic	Inherited (paternal)	Diagnostic	Familial platelet disorder; transplantation donor changed from sibling to unrelated donor	No

15-33031^c^	MDS	*GATA2* c.16delG (p.E6fs)	Heterozygous, frameshift	Pathogenic	*de novo*	Diagnostic	Supports transplant recommendation; genetic counseling for family	No

15-90485^c^	HLH	*C1QA* c.622C > T (p.Q208Ter)	Homozygous; nonsense	Pathogenic	Inherited (heterozygous parents)	Diagnostic	C1Q deficiency	No

14-19751^c^	HLH	*MLL2* c.11640_11640delG (p.M3881Cfs9); c.15631G > A (p.E5211K)	Compound heterozygous, frameshift; missense	Pathogenic	*de novo*; inherited (maternal)	Diagnostic	Kabuki syndrome; transplant withheld	No

15-46877^c^	HLH	*XIAP* c.1328G > C (p.R443P)	Hemizygous, missense	Likely pathogenic	Inherited (maternal)	Diagnostic	X-linked lymphoproliferative syndrome 2 (XLP2); transplant recommended	No

**ACMG secondary findings**								

14-20062^a^^,^^c^	B-ALL	*SDHC* c.224G > A (p.G75D)	Heterozygous, missense	Likely pathogenic	Inherited (maternal)	Health maintenance/genetic counseling	Predisposition for hereditary paraganglioma-pheochromocytoma	No

14-92247^c^	T-ALL	*PMS2* c.1376C > G (p.S459X)	Homozygous, nonsense	Pathogenic	Inherited (heterozygous parents)	Health maintenance/genetic counseling	Constitutional mismatch repair deficiency syndrome; Lynch syndrome	No

15-29224^a^^,^^c^	AML	*TP53* c.644G > A (p.S215N)	Heterozygous, missense	Likely pathogenic	Inherited (paternal)	Affects therapy/health maintenance/genetic counseling	Influenced choice for palliative therapy; increased risk for developing other cancers	No

16-17446^a^	AML	*RET* c.2410G > A (p.V804M); c.2832C > G (p.I944M)	Heterozygous, missense; heterozygous, missense	Pathogenic; VOUS	Inherited (paternal)	Health maintenance/Genetic counseling	Predisposition for familial medullary thyroid cancer, multiple endocrine neoplasia type 2A, and multiple endocrine neoplasia type 2B	No

*AML, acute myeloid leukemia; MDS, myelodysplastic syndrome; ALCL, anaplastic large cell lymphoma; HLH, hemophagocytic lymphohistiocytosis; B-ALL, B-cell acute lymphoblastic leukemia; VOUS, variant of unknown significance*.

*^a^Case listed more than once*.

*^b^Presumed inherited, parents were not tested*.

*^c^Case previously reported (11)*.

### Germline Findings and Clinical Impact in Pediatric Patients with Hematologic Disorders

Sequencing of germline tissue was performed in 50/56 patients. In six patients, only constitutional WES was performed, because of a strong suspicion of a cancer predisposing condition (*n* = 3) or an underlying condition predisposing to the development of hemophagocytic lymphohistiocytosis as described above (*n* = 3) (Table 1). Constitutional WES was requested in 2/6 patients based on clinical observations and in the absence of any family history suggestive of possible predisposition to cancer. One patient developed persistent thrombocytopenia following completion of planned chemotherapy for AML and was referred for stem cell transplant; mild thrombocytopenia was noted during the screening work-up of her donor sister leading to the suspicion of an underlying familial platelet disorder; constitutional WES identified a germline mutation in *RUNX1* leading to a change in donor selection and referral of the affected family members to the clinical genetic service. The second patient had persistent anemia and thrombocytopenia, but no definitive diagnosis was obtained from the work-up, which included analysis of the bone marrow aspirate and biopsy. Constitutional WES identified a *de novo* germline frameshift mutation in *GATA2* (56), thus explaining the clinical findings of peripheral blood cytopenias and leading to a recommendation for stem cell transplant. In the remaining 44 patients, germline tissue was sequenced through WES/RNA-seq. Germline findings were identified in 7/44 patients (16%) and included variants in three genes recommended for return by ACMG (Figure 3; Table 4). The three patients and their families were referred to a clinical geneticist for the return of results. Additionally, for one of these three patients, a 1-year-old girl with Down syndrome related AML refractory to chemotherapy, the finding of germline *TP53* mutation contributed to the early decision to proceed to palliative care rather than intensive therapy with curative intent. Importantly, the family history for these three patients was not suggestive of an inherited cancer syndrome and a referral to clinical genetics would have not been requested. Overall, clinically impactful germline variants were found in 12/50 patients (24%) (Table 4).

## Discussion

The majority of patients diagnosed with hematologic malignancies can now be cured. This remarkable success has been achieved through well-designed clinical trials conducted within large consortia. Refined risk stratifications and increasingly higher doses and more intense schedules of conventional chemotherapy have led to improved outcomes. However, oncologists recognize that further intensification of therapy would be unsafe and likely not effective. Moreover, the lack of understanding of the basic mechanisms of resistance to therapy has posed a limit to the ability to more precisely predict who would benefit from therapy and who should be spared from its side effects. This sentiment, together with an increasing number of successful examples of targeted therapy in pediatric and adult cancers, has led to much enthusiasm and support for a new wave of genomics driven pediatric oncology. Over the past decade, advances in genomics technology and computing, together with progress in drug discovery, have created a unique opportunity to test whether a broader knowledge of genomic alterations present in individual tumors can guide the choice of therapy and lead to improved outcome and overall superior care for the individual patient.

In this report, we described the feasibility of incorporating comprehensive genomic profiling into the care of pediatric patients with high-risk hematologic malignancies and blood disorders, and highlight how it influenced decision-making and overall clinical management in areas that go beyond therapeutics. Based on current knowledge of the common oncogenic drivers in pediatric hematologic disorders, we utilized whole exome and RNA sequencing to identify genomic alterations of relevance in the clinic. With this approach, we found that in 90% of patients, NGS findings fit our definition of “clinically impactful.” Targetable genomic alterations were identified in 80% of the patients. The high incidence of recurrent targetable mutations is likely due to the high-risk nature of the diseases tested. Unfortunately, only 12.5% of patients received targeted therapy. The difference between the incidence of targetable alterations and the actual implementation of targeted therapy is striking and in agreement with previous reports (12, 13). As expected, many pediatric patients are enrolled on clinical trials or are treated with well-established regimens for which combination with targeted therapies is not permitted or lacks safety data respectively. However, the most common reasons for the limited use of targeted therapies was the paucity of safety and efficacy data in pediatric tumors, limiting the motivation of treating physicians to offer them to patients. Additionally, the process for obtaining non FDA approved experimental drugs for pediatric patients is lengthy and access to drugs is rarely granted for younger patients. Awareness of these challenges is increasing and will hopefully lead to improved processes and expanded access to targeted therapies.

Of equal relevance to the finding of targetable alterations was the recognition that the knowledge of the genetic characteristics of each individual tumor improved overall clinical decision-making. This information together with the clinical history and the pathologic findings, guided the clinical management for 25% of patients. Challenging diagnoses were clarified, prognosis and risk classification were corrected, and the evolution of disease was elucidated through the analysis of serial samples, leading to decisions to intensify, simplify, or modify therapy (beyond the use of targeted drugs) that would have not been possible with routine available tests.

The inclusion of RNA-seq in our sequencing platform proved highly advantageous. Over 50% of identified fusions were targetable and 4/12 patients received effective targeted therapy based on their RNA-seq data. Furthermore, more than one-third of the fusions identified through RNA-seq were cryptic and had not been previously identified through karyotype and FISH analysis. For example, cryptic fusions such as *CBFA2T3-GLIS2* and *NUP98-NSD1*, which carry clear prognostic significance, were identified. The use of WES of paired tumor and normal samples revealed the presence of germline alterations that predispose to cancer in ~6% of patients. Importantly, the family history would have not prompted a referral to a genetic clinic during routine pediatric care. Interestingly, the majority of patients had agreed to the return of secondary findings and the referral for genetic counseling and further genetic evaluation was very well received.

Overall, we conclude that the combination of WES and RNA-seq for genomic profiling of pediatric hematologic disorders provide substantial information that can broadly impact clinical care in a meaningful way that is sufficient to justify its routine use at least for high-risk patients. While the promise of precision medicine has been based on the prospects that a precise knowledge of the genome of a patient’s tumor will lead to more rational therapeutic choices (57–59), it is clear that genome mining technologies, together with detailed clinical and histologic data, influences every step of clinical care, from time of diagnosis to delivery of therapy and after.

Our experience has also exposed several challenges to the implementation and expansion of precision medicine in pediatric oncology. Cost and turnaround time remain a major challenge; while the expenses for reagents and equipment are declining, it is expected that infrastructure and personnel costs will increase to accommodate storage and analysis of increasingly complex data. Molecular profiling of pediatric and adult cancers has exposed the complex structure of individual tumors, the dynamic changes that occur during therapy and the heterogeneity of patients’ tumors within the same class of diseases. Furthermore, more than one driver gene is frequently present in a tumor sample; only a few genes are effective targets; the role of specific genetic alterations in different tumor types is frequently unknown; and multiple combinations of different driver genes exist within the same diseases. All of these factors and many others are challenges that oncologists and teams dedicated to the analysis and interpretations of data, regularly face and hamper the effective clinical applicability of genomic profiling of tumors. In the future, establishment and continuous support for large clinical molecular databases, design of more powerful and ideally standardized analytical algorithms, novel clinical trial designs, support for teams of dedicated oncologists, bioinformaticians and scientists to interpret these data, and education of future generation of physicians are a few of the priorities to ensure that the promise of precision medicine is fulfilled.

## Ethics Statement

All participants provided written informed consent for tumor and/or germline sequencing analysis. Participants signed either a Columbia University Medical Center (CUMC) Institutional Review Board (IRB) approved consent (IRB# AAAB7109) or clinical consent; (https://pathology.columbia.edu/diagnostic/PGM/oncologytests.html). Our research was carried out in accordance with the Declaration of Helsinki and Good Clinical Practice (GCP). IRB approval was obtained for this retrospective analysis of de-identified patient and clinical genomics data (IRB AAAQ8170 and AAAP1200).

## Author Contributions

MS, LM, JO, JB and AK, designed and supervised all phases of the project. DP, AS, and RZ provided coordination of clinical samples and testing. MM and AS directed the clinical laboratory and SH oversaw sample preparation, data processing, and computational analysis. MM, SH, and AT carried out clinical analysis, interpretation, and reporting to the EMR. MS, JB, and AK provided clinical expertise and interpretation of clinical sequencing data at the multidisciplinary tumor board, and were responsible for overseeing consent and return of results. WC provided critical advice on the interpretation and return of germline findings and provided oversight of genetic counseling. CC provided genetic counseling. JP carried out data processing and computational analysis. JB, JO, and AK provided funding acquisition. MS, LM, and JO wrote the paper. All authors contributed to data interpretation, discussion, and editing of the paper. All authors read and approved the final manuscript and agreed to be accountable for all aspects of the work.

## Conflict of Interest Statement

The authors declare that the research was conducted in the absence of any commercial or financial relationships that could be construed as a potential conflict of interest.

## Footnotes

^1^https://pathology.columbia.edu/diagnostic/PGM/oncologytests.html.

^2^http://cbioportal.org.
