# Supplementary material for: Precision Medicine in Children and Young Adults with Hematologic Malignancies and Blood Disorders: The Columbia University Experience
Source: Front Pediatr. 2017 Dec 12;5:265. doi: 10.3389/fped.2017.00265 (PMC5732960; doi:10.3389/fped.2017.00265)
Supplement: Supplementary file 1 [file Table_1.PDF]

Supplementary Table S1. Quality statistics for whole exome sequencing

	Tumor (n = 63)				Normal (n = 47)			
	**AVG**	**STD**	**Pct_10th**	**Pct_90th**	**AVG**	**STD**	**Pct_10th**	**Pct_90th**
Total_Reads	191806310	46288350.69	140200362	247251784	186469432.9	37491506.61	146171421	230021577.5
Aligned_Reads	184939575.6	44520051.55	135460628.4	239237384	181832210.5	36260027.43	143658146.2	220987230
Reads_Ambiguous_Aligned[a]	184299471.7	44553490.21	135144098.2	238767972.8	181150703.4	36340712.43	143031127.8	220373078.6
Reads_on_Target	150189310.7	38126194.04	107696892	195877211	147620917.4	30476110	116242836.8	180890559.4
Maximum_Coverage	9817.92	5932.112	4243.6	18250.8	10151.66	5409.783	3634.4	18247
Average_Coverage	181.15	47.068	130.18	242.46	176.18	36.566	135.97	220.7
ROI_Percent_Covered	98.55	2.508	98.07	99.38	98.83	0.491	98.4	99.33
Indel_count	1020.33	527.219	825	1140.6	1132.43	156.421	963.4	1368
Missense_count	26355.67	3102.154	23506.8	29545.8	29213.06	3619.243	25837.6	33432.6
N_Ts	19289.71	1659.08	17507.60	21438.00	20918.64	2156.66	19047.00	23601.40
N_Tv	7045.29	1749.08	5950.60	8099.00	8262.40	1886.45	6724.00	12010.40
Ts/Tv	2.8	0.3	2.5	3	2.61	0.382	1.9	2.87
Low_Conf	2953.35	1597.479	2169.2	3899	5493.53	2891.568	3686.6	10015.2
Het	14906.67	3291.462	13022	17863.6	15580.19	3006.798	14021.4	18370.8
Hom	8289.76	1620.41	7904.8	9465	8550.06	1340.384	8210.4	9377.6
Hom/Het	0.57	0.086	0.47	0.65	0.56	0.073	0.47	0.62
LowConf/Het	0.2	0.098	0.14	0.24	0.35	0.168	0.24	0.63

Legend: ROI, region of interest. N_Ts, number of transition variants. N_Tv, number of transversions. Ts/Tv, transition to transvesion ratio. Het, heterozygous variant. Hom, homozygous variant.

[a]Output with “Allowable Ambiguous Alignments set to ‘0’.” It is the number of reads that mapped to a unique genomic loci.

Quality Control/ Assurance:

For whole exome sequencing, the uniformity of coverage is provided by the ROI percent covered, for which the requirement is 98% coverage. At this level of coverage, our analysis showed 10X coverage of 98.5%, and 30X coverage of 94.5% of the region of interest (180 million reads, which is our average). For whole exome, coverage of individual mutations is not output. For the targeted assay, at 500X, over 98% of the ROI is covered at least at 50X.
